# Limb Hypothermia for Preventing Paclitaxel-Induced Peripheral Neuropathy in Breast Cancer Patients: A Pilot Study

**DOI:** 10.3389/fonc.2016.00274

**Published:** 2017-01-10

**Authors:** Raghav Sundar, Aishwarya Bandla, Stacey Sze Hui Tan, Lun-De Liao, Nesaretnam Barr Kumarakulasinghe, Anand D. Jeyasekharan, Samuel Guan Wei Ow, Jingshan Ho, David Shao Peng Tan, Joline Si Jing Lim, Joy Vijayan, Aravinda K. Therimadasamy, Zarinah Hairom, Emily Ang, Sally Ang, Nitish V. Thakor, Soo-Chin Lee, Einar P. V. Wilder-Smith

**Affiliations:** ^1^Department of Haematology-Oncology, National University Health System, Singapore, Singapore; ^2^Singapore Institute for Neurotechnology, National University of Singapore, Singapore, Singapore; ^3^Department of Biomedical Engineering, National University of Singapore, Singapore, Singapore; ^4^Institute of Biomedical Engineering and Nanomedicine, National Health Research Institutes, Zhunan Township, Taiwan; ^5^Department of Medicine, National University Health System, Singapore, Singapore; ^6^Neurology Diagnostic Laboratory, National University Hospital, Singapore, Singapore; ^7^National University Cancer Institute, National University Health System, Singapore, Singapore; ^8^Department of Biomedical Engineering, Johns Hopkins University, Baltimore, MD, USA; ^9^Cancer Science Institute of Singapore, National University of Singapore, Singapore, Singapore; ^10^Department of Medicine, Yong Loo Lin School of Medicine, National University of Singapore, Singapore, Singapore

**Keywords:** chemotherapy-induced peripheral neuropathy, limb hypothermia, paclitaxel, nerve conduction, neuroprotection

## Abstract

**Background:**

Peripheral neuropathy (PN) due to paclitaxel is a common dose-limiting toxicity with no effective prevention or treatment. We hypothesize that continuous-flow limb hypothermia can reduce paclitaxel-induced PN.

**Patients and methods:**

An internally controlled pilot trial was conducted to investigate the neuroprotective effect of continuous-flow limb hypothermia in breast cancer patients receiving weekly paclitaxel. Patients underwent limb hypothermia of one limb for a duration of 3 h with every paclitaxel infusion, with the contralateral limb used as control. PN was primarily assessed using nerve conduction studies (NCSs) before the start of chemotherapy, and after 1, 3, and 6 months. Skin temperature and tolerability to hypothermia were monitored using validated scores.

**Results:**

Twenty patients underwent a total of 218 cycles of continuous-flow limb hypothermia at a coolant temperature of 22°C. Continuous-flow limb hypothermia achieved mean skin temperature reduction of 1.5 ± 0.7°C and was well tolerated, with no premature termination of cooling due to intolerance. Grade 3 PN occurred in 2 patients (10%), grade 2 in 2 (10%), and grade 1 in 12 (60%). Significant correlation was observed between amount of skin cooling and motor nerve amplitude preservation at 6 months (*p* < 0.0005). Sensory velocity and amplitude in the cooled limbs were less preserved than in the control limbs, but the difference did not attain statistical significance. One patient with a history of diabetes mellitus had significant preservation of compound muscle action potential in the cooled limb on NCS analysis.

**Conclusion:**

This study suggests that continuous limb hypothermia accompanying paclitaxel infusion may reduce paclitaxel-induced PN and have therapeutic potential in select patients and warrants further investigation. The method is safe and well tolerated.

## Introduction

Chemotherapy-induced peripheral neuropathy (CIPN) is a common dose-limiting toxicity of paclitaxel. At present, dose modification remains the most successful approach for the management of CIPN, and pharmacological treatment is limited to alleviating symptoms such as paresthesias, dysesthesia, and pain (1). To date, none of the potential neuroprotective agents tested in clinical trials have proven effective (2).

The mechanisms of neurotoxicity in paclitaxel-induced peripheral neuropathy (PN) have not been fully elucidated; however, disruption of microtubule dynamics has been identified. Taxanes binding to β-tubulin components of microtubule assemblies lead to microtubule stabilization, thereby causing a disruption of microtubule dynamics (3, 4). It has also been observed that paclitaxel administration produces abnormalities in axonal mitochondria (5). Additional targets of neurotoxicity include direct axonal toxicity at the distal nerve terminals (6). Patients with pre-existing conditions that are capable of inducing PN (such as diabetes, Charcot–Marie–Tooth, or kidney disease) are particularly predisposed to developing CIPN (7).

Given the dose-dependent pathophysiology of paclitaxel-induced PN, we proposed a novel strategy for prevention of paclitaxel-induced PN by employing continuous-flow limb hypothermia to reduce delivery of the toxic chemotherapeutic agents to the peripheral nerves. Our previous *in vivo* study showed that a drop in rat sciatic nerve temperature from 30 to 20°C produced a fivefold reduction of nerve blood flow (8). Furthermore, in studies of chemotherapy-induced alopecia (CIA), which is a result of toxic accumulation of chemotherapeutics in the hair follicle, there is compelling evidence that cooling of the scalp protects against the development of CIA (9, 10). The rationale behind using hypothermia in the prevention of CIA is that scalp cooling decreases the blood supply to the hair follicles, and hence, hair follicle protection is a result of reduced delivery of toxic chemotherapeutics (10). However, scalp cooling employing traditional cooling methods such as ice packs is poorly tolerated, which limits efficacy of the treatment itself (11). Hence, we employed a better-tolerated and efficient cooling technique of continuous-flow hypothermia. In a previous study in healthy subjects, we also established that continuous-flow limb hypothermia at a coolant temperature of 22°C was the lowest tolerable temperature for a duration of 3 h, matching the duration of paclitaxel infusion in cancer patients (12).

The goal of the current study was to determine if continuous-flow limb hypothermia may be neuroprotective in patients receiving paclitaxel chemotherapy, as well as assessing safety and tolerability.

## Patients and Methods

### Study Design

**Figure 1:**
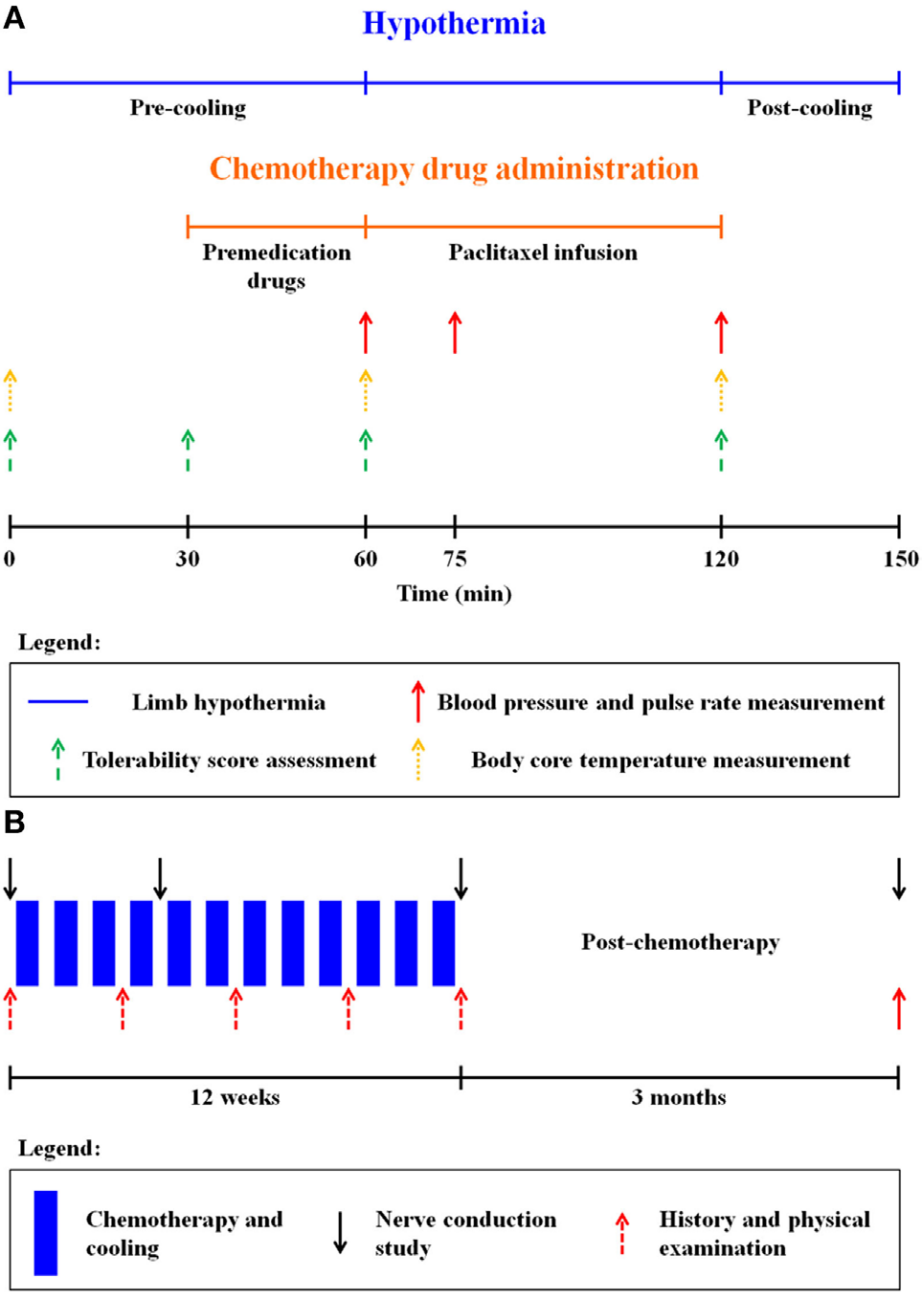
**(A)** Limb hypothermia protocol for one chemotherapy cycle. Premedication drugs: dexamethasone, diphenhydramine, and ranitidine. **(B)** Study schema.

**Figure 2:**
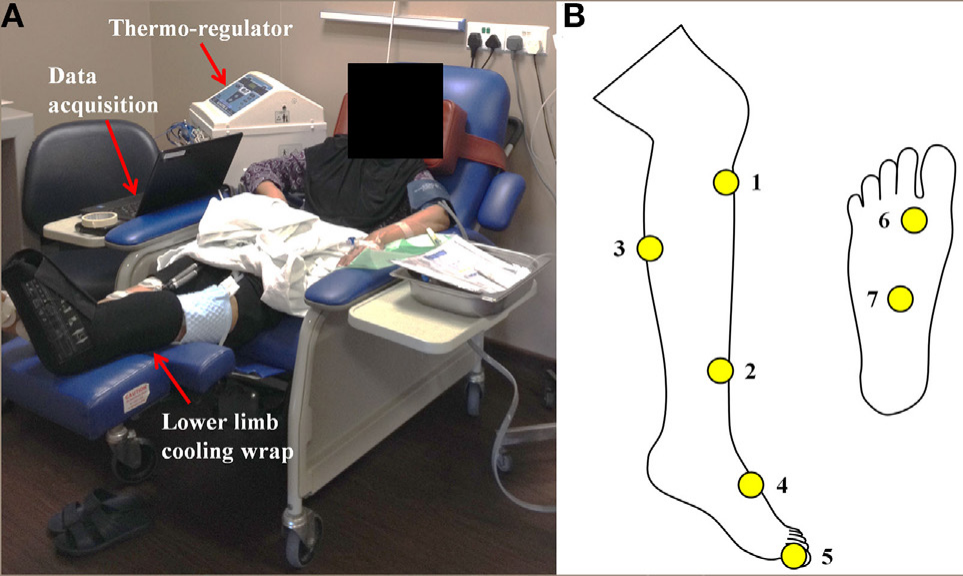
**(A)** Continuous-flow limb hypothermia setup by means of a thermoregulator device supplying coolant (water) at a set desired temperature (22°C) to limb wraps that cool the limb. Continuous skin temperature data are acquired *via* a temperature monitoring system consisting of wireless sensors placed at seven different sensor locations on the cooled and control legs as indicated in **(B)**, which transmit data wirelessly to a receiver and recorded for analysis.

This prospective study was carried out in accordance with the recommendations of the Institutional Review Board of the National Health Group, Singapore, with written informed consent from all subjects. All the subjects gave written informed consent in accordance with the Declaration of Helsinki. The study population comprised breast cancer patients scheduled to receive adjuvant weekly paclitaxel chemotherapy for 12 cycles following standard anthracycline-based chemotherapy (doxorubicin and cyclophosphamide). (For detailed inclusion/exclusion criteria, see supplementary material.) During every cycle of chemotherapy, premedication drugs (dexamethasone, diphenhydramine, and ranitidine) were administered 30 min prior to paclitaxel infusion. 80 mg/m^2^ of paclitaxel was administered as a 1-h infusion (indicated in orange in Figure 1A). The chemotherapy unit ambient temperature was adjusted to 21°C *via* air-conditioning. Randomization for limb cooling was carried out and the non-cooled limb served as internal control prior to the first cycle of therapy, and the same limb underwent cooling for all subsequent cycles, while the non-cooled limb remained as control (Figure 2A).

Limb hypothermia sessions comprised of a pre-cooling period (1 h), continued with paclitaxel infusion and a post-cooling period (on average 30 min after the end of paclitaxel infusion) (Figure 1A). Overall, hypothermia was administered for no longer than 4 h. A detailed safety protocol was followed for coolant thermoregulation, if the patient found the hypothermia intolerable (Tables S1 and S2 in Supplementary Material).

Safety and tolerance of limb hypothermia were measured using three validated scales: visual analog pain scale (VAS), subjective tolerance scale, and the Shivering Assessment Scale (Figure S1 and Tables S3 and S4 in Supplementary Material) (13, 14). Skin surface temperature was continuously recorded throughout limb hypothermia *via* temperature sensors (accurate to ±0.1°C) placed at seven locations on both the legs (Figure 2B) (12). Body core temperature was measured over the frontal non-glabrous scalp (Figure 1A).

### Assessment of Neuropathy

Assessment for neuropathy was performed using nerve conduction studies (NCSs) and clinical examination. NCSs are the most sensitive and specific detection method for neuropathies and superior to clinical examination or scores (15). Primary endpoint was differences in NCSs carried out at baseline (NCS_baseline_), 1 month into treatment (NCS_mid_), the end of treatment (NCS_end_), and 3 months post-treatment (NCS_3m_) (Figure 1B). Sensory nerve action potential (SNAP) amplitudes and conduction velocities were measured in the bilateral sural, superficial peroneal, saphenous, and medial and lateral plantar nerves (16). Compound motor action potential (cMAP) amplitudes and motor nerve conduction velocities were evaluated in the bilateral common peroneal and tibial nerves (17).

At the same time points, clinical evaluation using the validated Total Neuropathy Score (TNS) was performed (18).

### Statistical Analysis

Temporal trend of skin temperature variation over the duration of hypothermia was summarized as an average of the recorded temperatures for all cycles of cooling for all the patients. Similarly, tolerability was analyzed as an average of all patients’ tolerance scores across all cycles of cooling. Sensory and motor nerve parameters of amplitude and velocity at every NCS visit were analyzed as relative percentage changes with respect to the first NCS visit (NCS_base_) and averaged across patients.

We assessed the effect of varying amounts of cooling on nerve conduction parameters through correlation analysis (Pearson). Limb cooling was quantified by calculating each patient’s average reduction in baseline skin temperature over 12 cooling cycles. Limb cooling was correlated with mean SNAP amplitude/velocity percentage changes at the sural, superficial peroneal, and saphenous nerves. Similarly, limb cooling was correlated with mean cMAP amplitude/velocity percentage changes from all peroneal nerve stimulation points (ankle, below fibula head, and above fibula head) at the recording site of the extensor digitorum brevis (EDB) and from the tibial nerve ankle stimulation point on the abductor hallucis. This was done for values obtained at NCS_end_ and NCS_3m_. A negative correlation shows that more cooling results in better preservation of nerve conduction parameters. Comparison of three different degrees of cooling achieved and the relation to the degree of preservation on nerve conduction parameters were also assessed.

Continuous variables are shown as mean ± SD. A parametric paired *t*-test was used to compare the changes in temperature and NCS values of the cooled and control limbs in each patient. A two-tailed *p*-value <0.05 was considered statistically significant. The Pearson’s correlation coefficient was calculated to determine the correlation between amount of limb cooling and NCS preservation. All statistical analyses were performed in Microsoft Excel (V.12.0 for Windows, Microsoft Corp., Washington, DC, USA).

## Results

Twenty female breast cancer patients were enrolled in the study (Table 1). Of these 20 patients, 17 (85%) completed 12 cycles of continuous-flow limb hypothermia and one patient developed an infected seroma after her ninth cycle and deemed not fit for further paclitaxel by the treating oncologist. The abovementioned 18 patients completed all TNS and nerve conduction assessments before and after chemotherapy and were included in the analysis of nerve conduction changes and assessment of clinical neuropathy. The remaining two patients who were enrolled in the study did not complete all assessments and hence were not included for analysis of nerve conduction changes and assessment of clinical neuropathy [one patient completed two cycles before discontinuing due to development of grade 3 PN. Another withdrew from the study after three cycles due to ineligible inclusion criteria (not adjuvant therapy, Stage IV disease)]. However, all the 20 enrolled patients were included for safety and tolerability analysis.

**Table 1:** **Baseline patient characteristics**.

Variables	*N* (%) (total *N* = 20)	Mean (range)
Age (years)		53 (32–67)
Weight (kg)		60 (38–81)
Height (cm)		154 (135–167)
BSA (m^2^) baseline		1.6 (1.2–1.9)
Cumulative dose of paclitaxel (mg/m^2^)		868.0 (160.0–960.0)
Cancer stage		–
Stage 1	3 (15)	
Stage 2	11 (55)	
Stage 3	5 (25)	
Stage 4	1 (5)	
Type of surgery		–
Breast conservation	5 (25)	
Mastectomy	15 (75)	
Lymph node assessment		–
Sentinel lymph node biopsy	9 (45)	
Axillary clearance	8 (40)	
Both	3 (15)	
ER-positive	18 (90)	–
Her-2-positive	3 (15)	–
Concurrent herceptin	3 (15)	–
TNS baseline		–
0	15 (75)	
1	4 (20)	
2	1 (5)	

### Safety and Tolerability

Continuous-flow limb hypothermia was well tolerated by all patients. Premature termination of cooling was never necessary and only one patient (for 2 out of a total 218 cycles) required one intra-cycle thermoregulator temperature increase of 1°C toward the end of a hypothermia session (Figure S2 in Supplementary Material). Overall, minimal discomfort was reported at the end of each limb hypothermia session. No serious or lasting adverse events as a result of hypothermia were encountered. Only temporary erythema lasting a few minutes was observed upon removal of the cooling wrap. All recorded adverse events were due to chemotherapy (Table S5 in Supplementary Material). Patients’ core body temperature showed negligible changes (0.03 ± 0.18°C) across chemotherapy cycles.

### Skin Temperature Changes with Limb Hypothermia

**Figure 3:**
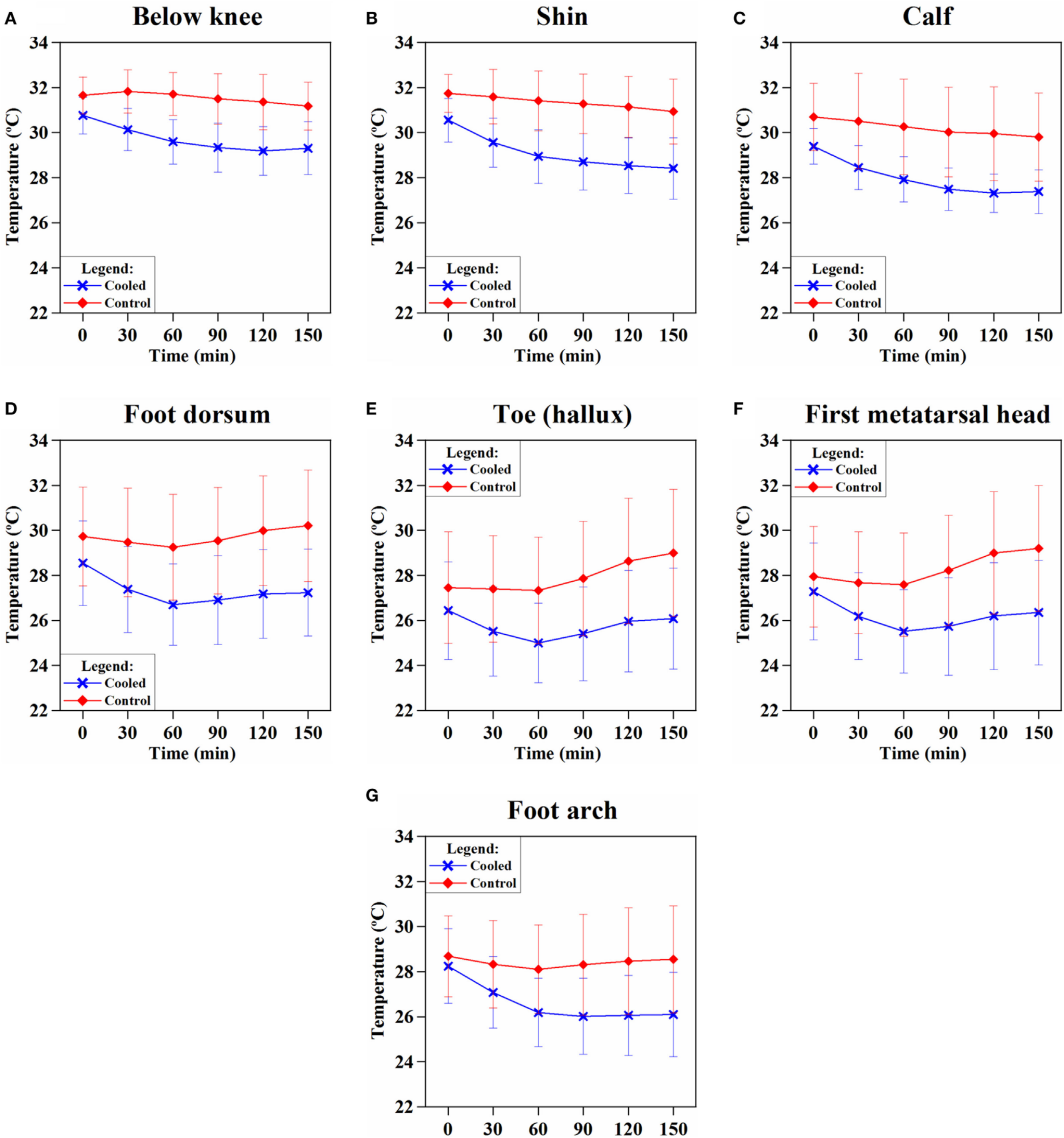
**Trend of skin temperature of the cooled (blue) vs. control (red) leg over the duration of limb hypothermia in breast cancer patients**. Skin temperature was acquired continuously at various sensor locations: **(A)** below knee, **(B)** at the shin, **(C)** calf, **(D)** dorsum of the foot, **(E)** toe (hallux), **(F)** first metatarsal head, and **(G)** foot arch (foot plantar). Skin temperatures of the cooled leg showed significantly lower temperatures than the control leg at each time point (*p* < 0.05). Limb hypothermia was administered at a coolant temperature of 22°C throughout the duration of chemotherapy.

Skin temperature changes at all the seven sensor locations on the cooled and control limbs were calculated and averaged across all patients over all 218 cycles. Following the onset of hypothermia, skin temperatures of the cooled leg showed significantly lower temperatures than the control leg (*p* = 0.0003) (Figures 3A–G). A mean temperature drop of 1.5 ± 0.7°C was achieved across all sensor points on the cooled limb and averaged across all the patients. The largest temperature drops in the cooled limb was achieved in the shin (2.2 ± 1.1°C) (Figure 3B) and foot arch (2.2 ± 1.3°C) (Figure 3G).

### Clinical Neuropathy

Assessment of neuropathy using clinical and nerve conduction parameters of 18 patients was done. The TNS grade of PN reported during all the four visits were documented (Figure S3 in Supplementary Material). Baseline TNS ranged between 0 and 2 for all patients, thereby indicating absence of any baseline neuropathy. As per the National Cancer Institute-Common Toxicity Criteria grading of neuropathy (19), sensory PN of the following grades were experienced by patients at the end of chemotherapy: grade 3 PN occurred in two patients (10%), grade 2 in two patients (10%), grade 1 in 12 patients (60%), and grade 0 in four patients (20%). One patient developed grade 3 PN after two cycles and dropped out of the study. She was included for safety analysis but not efficacy analysis. The other patient with grade 3 PN completed all 12 cycles. Both patients with grade 2 PN completed 12 cycles of limb hypothermia.

### Nerve Conduction Changes

**Figure 4:**
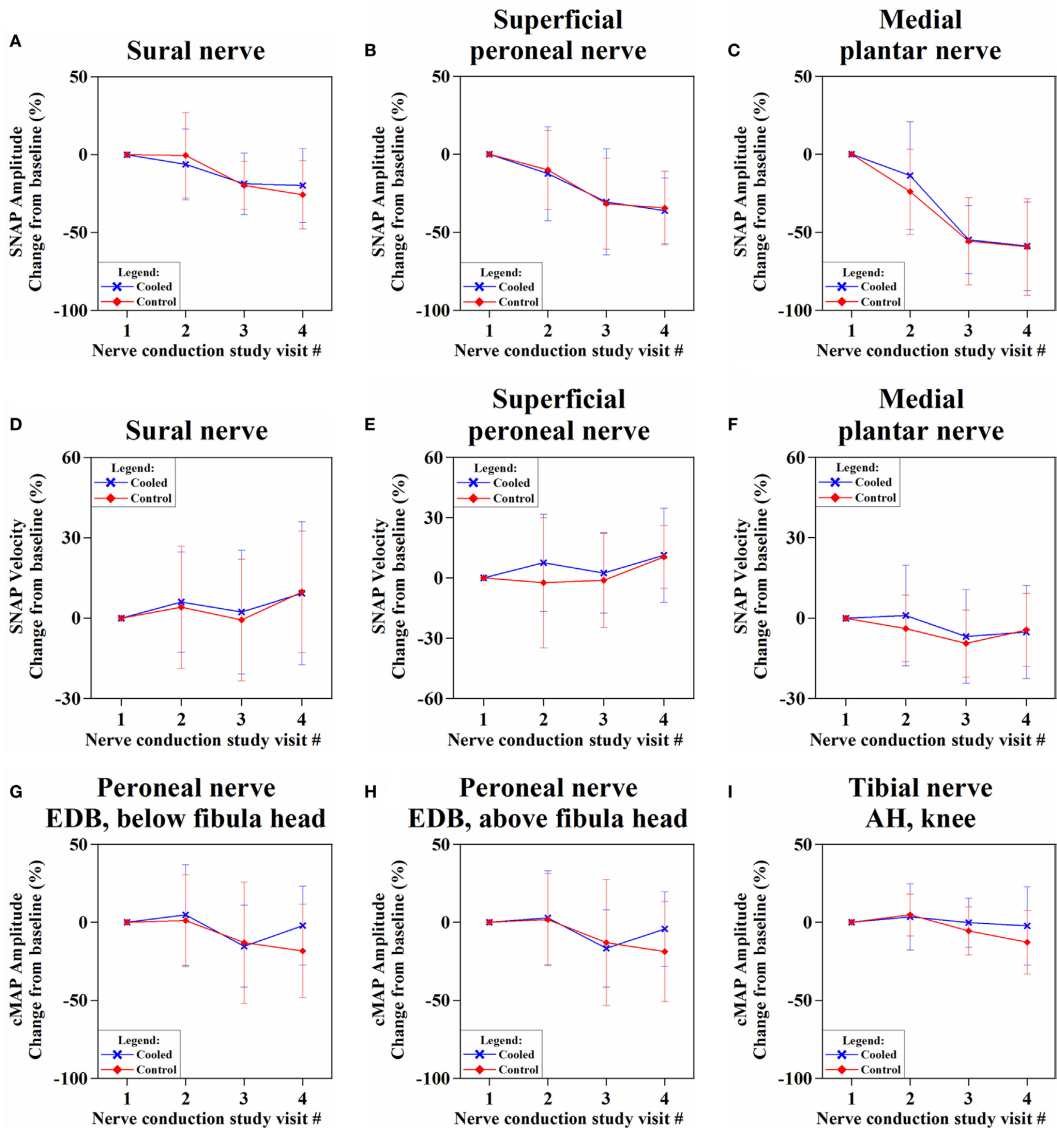

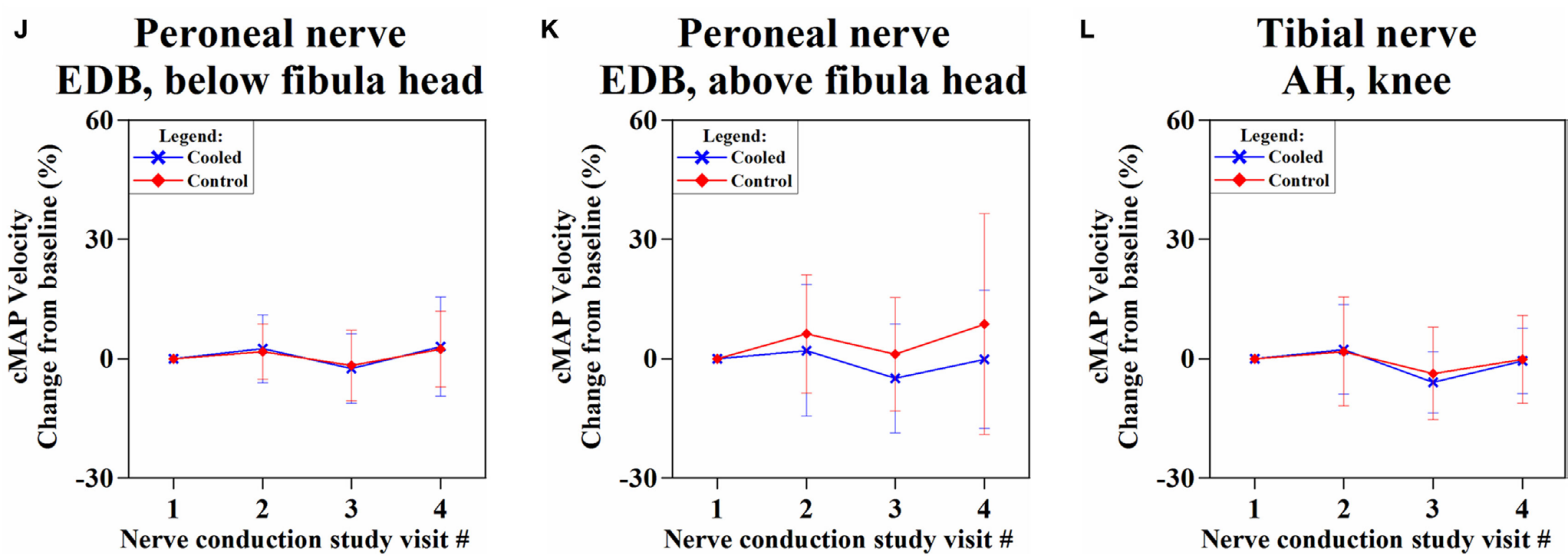
**Percentage change of sensory nerve action potential (SNAP) amplitudes (A–C) and velocities (D–F), and compound motor action potential (cMAP) amplitudes (G–I) and velocities (J–L) over four nerve conduction study visits from baseline**.

The SNAP amplitudes showed decreasing trend over time, in both cooled and control limbs (Figures 4A–C; Table S6 in Supplementary Material). While the sural nerve showed more preservation of SNAP amplitude in the cooled than in the control limb at NCS_3m_, the difference was not significant [−19.9 ± 23.7% (cooled) vs. −25.8 ± 21.8% (control), *p* = 0.16]. Sensory velocities between the limbs showed no significant difference (0.09 < *p* < 0.89) (Figures 4D–F; Table S6 in Supplementary Material).

The cMAP amplitudes of all recorded motor nerves were more preserved in the cooled limb than the control limb (Figures 4G–I; Table S7 in Supplementary Material) at NCS_3m_, without reaching significance. At NCS_3m_, the EDB cMAP amplitudes of the cooled limb showed more preservation [stimulation below fibula head: −2.1 ± 25.3% (cooled) vs. −18.3 ± 30.0% (control), *p* = 0.07; stimulation above fibula head: −4.3 ± 23.9% (cooled) vs. −18.7 ± 32.0%, *p* = 0.10] (Figure 4G). Motor velocities did not show significant change between limbs (Figures 4J–L; Table S7 in Supplementary Material).

**Figure 5:**
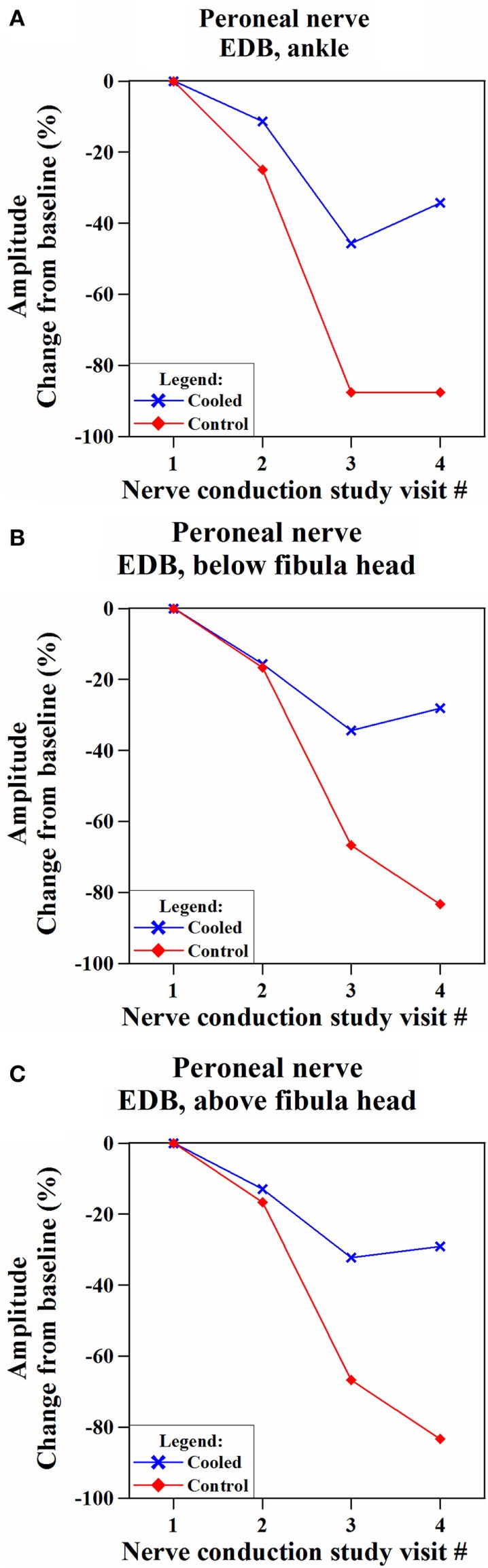
**Comparison of changes in compound motor action potential (cMAP) amplitudes in the cooled and non-cooled limb of a subject with well-preserved cMAP amplitudes in the extensor digitorum brevis (EDB) muscle on the common peroneal nerve**. Over the time, the cooled leg showed consistently more preserved compound motor action potential amplitudes than the control leg at all the three stimulation points [**(A)** ankle, **(B)** below fibula head, and **(C)** above fibula head] on the EDB muscle.

We identified one subject who experienced significant preservation of cMAP amplitudes (EDB) in the cooled leg, compared to the control leg (Figure 5). This was a 64-year-old female patient with Stage II hormone receptor-positive and Her2-positive breast cancer. She had a history of diabetes with an Hba1c of 7.3% at the time of her screening visit. She had no pre-existing neuropathy from her diabetes with a baseline TNS score of 1.

While her cMAP (EDB) amplitudes in the cooled and control legs were below baseline for NCS_mid_, NCS_end_, and NCS_3m_, separation between cMAP in the cooled and control legs was shown at NCS_end_ where cMAP amplitude (EDB, ankle stimulation) was 41.8% higher in the cooled leg than the control leg. At NCS_3m_, cMAP amplitude (EDB, ankle stimulation) was 53.2% higher in the cooled leg.

### Effect of the Amount of Limb Cooling on NCS

The greatest negative correlation was between cooling and cMAP recordings over the EDB with distal stimulation at NCS_3m_ (*r* = −0.55). SNAP amplitudes and limb cooling did not show a large correlation at NCS_end_ (*r* = −0.17) or NCS_3m_ (*r* = −0.13).

**Figure 6:**
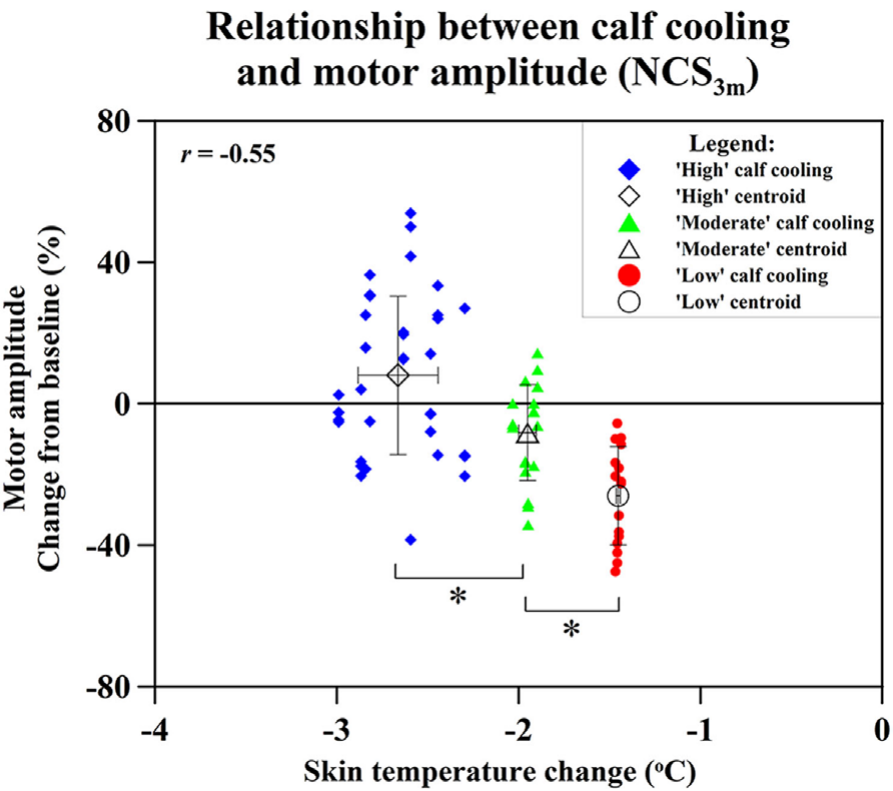
**Relation of changes of skin temperature at the calf and compound motor action potential (cMAP) amplitude percentage changes of extensor digitorum brevis (distal stimulation) cMAP at NCS_3m_, with data grouped into “high” cooling, “moderate” cooling, and “low” cooling**. Statistical significance (*p < 0.05).

To ascertain whether different degrees of cooling achieved different preservation of cMAP amplitude, three different degrees of cooling were plotted against the achieved preservation in cMAP. All groups showed highly significant differences: high (Δ*t* > −2.2°C) vs. moderate cooling (Δ*t* 2.2–1.5°C) (*p* = 0.002); moderate vs. low cooling (Δ*t* < −1.5°C) (*p* = 0.0006) (Figure 6).

## Discussion

Our study shows that continuous-flow limb hypothermia using coolant temperatures of 22°C lasting the duration of paclitaxel chemotherapy is well tolerated and safe. Limb hypothermia with higher degrees of cooling significantly preserves selected nerve motor amplitudes at 3 months after start of chemotherapy.

### Safety and Tolerability of Continuous-Flow Limb Hypothermia

Our results show good tolerability (Figure S2 in Supplementary Material) and, importantly, no early termination of cooling.

Various limb cooling modalities are used for different therapeutic interventions, most of which involve the direct application of ice or frozen gloves and cause steep cooling gradients with varied and often poor tolerability (20). Large cooling gradients permit only intermittent coolant application and are limited by significant intolerance and sometimes frostbite (21, 22). Advantages of continuous-flow limb cooling with thermoregulation features are controlled and more tolerable temperature reduction for the duration of chemotherapy infusion with better outcomes (21). Concern could be raised regarding cold-induced nerve damage, which is known to occur, depending on length and degree of cooling. The work by Jia et al. has examined in detail the required circumstances for cold-induced ischemia to occur in animal experiments (23). It is concluded that cooling the sciatic nerve for 3 h at 2°C does not result in vascular occlusions or morphological change in the nerves suggestive of ischemia from vasoconstriction. Looking at the difference in temperatures it is unlikely that 22°C cooling will be able to result in additional counteractive ischemia.

### Skin Temperature and Hypothermia

Limb hypothermia caused significant decrease in skin temperature in the cooled limb across all sensor locations (Figure 3), while body core temperature was unaffected. The majority of cooling occurred in the first 60 min, consistent with other studies (24). Taking into consideration the objective of inducing maximal vasoconstriction before the initiation of chemotherapy, a period of pre-cooling, may be crucial (25). Hypothermia-induced vasoconstriction should be maintained throughout the duration of chemotherapy (12). All of these conditions were met by our study.

In studies of scalp cooling to prevent CIA, subcutaneous scalp temperature (depth 1–2 mm) had to be less than 22°C to prevent doxorubicin-induced alopecia (26). To achieve a subcutaneous temperature of 22°C, a surface scalp temperature of 19°C was necessary (27). Our study only achieved a mean skin surface temperature of 26.0 ± 0.8°C on the cooled limb. Although the targeted tissues are different, the temperature achieved in our study seems modest to achieve neuroprotection. We utilized a coolant temperature of 22°C based on this representing maximum tolerability for 3 h of continuous cooling (12). Future studies will need to explore techniques such as cryocompression to achieve more cooling while ensuring tolerability (28).

Although thermoregulator temperature was constant (22°C), different patients achieved different amounts of limb cooling due to varying body surface area and factors beyond experimental control, such as intermittent toilet breaks. On average, each patient took one toilet break per chemotherapy cycle typically lasting 5–7 min, during which limb wraps were removed. Toilet breaks for trial subjects were similar in nature to chemotherapy patients not undergoing limb hypothermia with respect to frequency and duration and were not likely increased due to hypothermia. These breaks were mostly immediately before or after the end of paclitaxel infusion itself (Figure 1A).

### Changes in Sensory and Motor Nerve Conduction Parameters

Both SNAP and cMAP amplitudes represent the number of functioning axons within each nerve. Lower amplitude reflects chemotherapeutic axonal damage, whereas reduction in velocity signifies nerve myelin sheath dysfunction (29, 30). Our results concur with the literature in showing paclitaxel-induced PN is predominantly axonal, length dependent, and sensory predominant (1).

Our study is the first to systematically record motor function over the course of chemotherapy using sensitive parameters (cMAP) and reveals frequent motor involvement. Clinical examination used in clinical scoring (TNS) was not able to detect motor involvement. Likely, this is because of poor sensitivity which explains the low rates of involvement in the literature (31).

It is important to consider why motor nerve parameters (cMAP) showed more effect to hypothermia than sensory parameters. cMAP amplitude, in contrast to sensory parameters, is dependent not only on nerve axonal function but additionally on muscle fiber function. Considering that paclitaxel motor neuropathy is rare (32) and myopathy is poorly identified with the TNS examination utilized, we suggest much of cMAP change could be from paclitaxel-induced myopathy. The infrequent reporting of myopathy in the literature is likely due to poor methods of detection (33).

Our hypothesis for the prevention of CIPN suggests that hypothermia reduces paclitaxel delivery to the nerve. Since hypothermia induces reduced blood flow to all exposed tissues, both motor axonal and myopathic components could underlie cMAP preservation (34). We postulate that prevention of myopathy may play a bigger role since hypothermia will affect muscle more than nerve based on the greater abundance of muscle tissue and the absence of a nerve–blood barrier.

Overall, our primary endpoint of differences in nerve conduction parameters between cooled and non-cooled limbs did not reach significance (Figure 4). Considering the modest degree of limb cooling achieved, we were particularly interested in assessing the effect of different degrees of cooling achieved. A good correlation (*r* = −0.55; *p* < 0.001) was found between the amount of cooling and the mean cMAP amplitude percentage changes at 3 months after the end of chemotherapy. This suggests that the degree of limb hypothermia achieved is crucial to the amount of cMAP preservation. Cluster analysis between high, moderate, and low amounts of cooling further showed significantly different amounts of cMAP amplitude preservation (Figure 6). This corresponds to observations suggesting a minimal threshold of temperature reduction for the prevention of alopecia (26). One patient benefited significantly from limb hypothermia, reflected through her NCS analysis. Several factors may have influenced this result including her history of diabetes, genetic predisposition, or concomitant medications. While sub-group analysis for these characteristics were not possible due to limited numbers, analysis of a larger cohort of patients undergoing limb hypothermia may identify predictive biomarkers for those who may benefit from such therapy.

Our study is adversely affected by the relatively small sample size and clinical methods that are not sensitive for assessment of mild or moderate degrees of motor dysfunction. Furthermore, only relatively modest degrees of limb cooling were achieved. While the lack of formal quality-of-life analysis in our study is a limitation, it was partially replaced by our safety scoring systems which did not reflect any concern. Future studies will also need to include more specific tests to determine the thresholds for mechanical and temperature sensitivity, or muscle strength, and to reveal alterations in sensory and motor functions related to neuropathy and myopathy (including plasma markers), as well as quality-of-life measures. A larger study using cryocompression of all four limbs is currently underway to prove efficacy. Studies to detect predictive biomarkers for paclitaxel-induced PN and to potentially identify patients from these protective therapies are also being conducted (35). Animal studies are also being performed to establish proof of reduced delivery of chemotherapy to neurons through limb hypothermia.

In summary, our results suggest that limb hypothermia, given for the duration of paclitaxel chemotherapy, preserves certain nerve conduction parameters. Preservation is directly related to the degree of limb cooling.

## Conclusion

Our findings open up a new opportunity for more research to be conducted toward the goal of achieving neuroprotection and preventing CIPN *via* a simple, non-invasive, and non-pharmacological method. As neuropathy is an important factor leading to chemotherapy dose reduction and treatment discontinuation, this research may contribute to alleviating dose limitation and increase the likelihood of success of chemotherapy. While our results revealed some interesting findings, it must only be regarded as a pilot study and larger studies achieving well tolerated and greater limb cooling are now needed.

## Author Contributions

RS, AB, NT, S-CL, and EW-S developed the study concept and design. RS, NK, AJ, SO, JH, DT, and JL treated patients in this trial. AB, ST, JV, AT, ZH, EA, SA, and EW-S were involved in data acquisition. RS, AB, ST, and EW-S analyzed the data. RS, AB, ST, L-DL, and EW-S drafted the manuscript. NT, S-CL, and EW-S supervised the study. RS and AB contributed equally to this work. This manuscript has been seen, read, and agreed upon in its content by all the designated authors.

## Conflict of Interest Statement

The authors declare that the research was conducted in the absence of any commercial or financial relationships that could be construed as a potential conflict of interest.

## Abbreviations

AH, abductor hallucis; CIPN, chemotherapy-induced peripheral neuropathy; cMAP, compound muscle action potentials; CTS, composite tolerability score; EDB, extensor digitorum brevis; NCI-CTC, National Cancer Institute-Common Toxicity Criteria grading of neuropathy; NCSs, nerve conduction studies; PN, peripheral neuropathy; PPMC, Pearson’s Product Moment Coefficient; SAS, Shivering Assessment Scale; SNAP, sensory nerve action potential; STS, subjective tolerance scale; TNS, Total Neuropathy Score; VAS, visual analog pain scale.
