# Supplementary material for: Limb Hypothermia for Preventing Paclitaxel-Induced Peripheral Neuropathy in Breast Cancer Patients: A Pilot Study
Source: Front Oncol. 2017 Jan 10;6:274. doi: 10.3389/fonc.2016.00274 (PMC5222823; doi:10.3389/fonc.2016.00274)
Supplement: Supplementary file 1 [file Data_Sheet_1.DOCX]

Supplementary Material

Limb Hypothermia for Preventing Paclitaxel-Induced Peripheral Neuropathy in Breast Cancer Patients: a pilot study

**Raghav Sundar**^†^**, Aishwarya Bandla**^†^**, Stacey Sze Hui Tan, Lun-De Liao, Nesaretnam Barr Kumarakulasinghe, Anand D. Jeyasekharan, Samuel Guan Wei Ow, Jingshan Ho, David Shao Peng Tan, Joline Si Jing Lim, Joy Vijayan, Aravinda K. Therimadasamy, Zarinah Hairom, Emily Ang, Sally Ang, Nitish V. Thakor, Soo-Chin Lee, and Einar P. V. Wilder-Smith^*^**

^†^The authors have contributed equally to this work.

**^*^Correspondence:** Professor Einar P. V. Wilder-Smith: einar_wilder-smith@nuhs.edu.sg

# Supplementary Data

## Inclusion/Exclusion Criteria

The inclusion/exclusion criteria were designed as follows. The study population comprised of breast cancer patients who were eligible for recruitment if they fulfilled the following criteria: i) aged 21-80 years; ii) histologically confirmed carcinoma of the breast; iii) scheduled to receive adjuvant weekly paclitaxel chemotherapy; and iv) signed informed consent from patient or legal representative. Patients were ineligible for the study if they had: i) open skin wounds or ulcers of the lower extremity; ii) pre-existing diagnosis of polyneuropathy; iii) medical contraindications for paclitaxel or taxane-based therapy; iv) a score of more than five in the TNS at baseline; or v) history of Raynaud’s phenomenon, peripheral vascular disease, or poorly controlled diabetes (HbA1c > 10%).

## Devices Used

The thermoregulator used in the study was the Blanketrol III (Cincinnati Sub-zero, OH, USA) together with VitalWear cooling wrap (VitalWear, San Francisco, CA, USA). The limb skin temperature sensors used were skin surface probes SST-1 (Physitemp, USA).

## Nerve Conduction Studies

Tests were performed as previously standardized; for the bilateral sural, superficial peroneal, saphenous, medial and lateral plantar nerves ([Karandreas et al., 1995](#_ENREF_1)) and for the bilateral common peroneal and tibial nerves ([Ping Ng et al., 2013](#_ENREF_2)).

# Supplementary Tables

**Table S1.** Protocol for thermoregulation within each cycle of chemotherapy, based on scores of dose limiting tolerability of patient cohorts.

| **Event** | **Action** |
| --- | --- |
| STS ≥ 2 | Coolant temperature to be increased by 1°C (up to a maximum coolant temperature of 25°C) every 15 minutes, until an STS of 1 is reached. |
| STS ≥3 | Coolant temperature to be increased 2°C (up to a maximum coolant temperature of 25°C) every 15 minutes, until an STS of 1 is reached. |
| CTS = Intolerable | Limb hypothermia to be terminated. |

**Table S2.** Protocol for thermoregulator coolant temperature determination for subsequent cycles.

| **Event** | **Action** |
| --- | --- |
| The patient tolerated at least 1 hour of cooling at the initial temperature of the previous cycle | The same initial temperature will be used for the subsequent cycle |
| Patient did not tolerate at least 1 hour of cooling at the initial temperature of the previous cycle | The initial temperature will be 1°C higher for the subsequent cycle |

**Table S3.** Subjective tolerance scale for assessing tolerability of continuous-flow limb hypothermia.

| **Subjective tolerance scale (STS)** | **Indications** |
| --- | --- |
| 0 | Tolerated no problems (no significant numbness) |
| 1 | No problem maintaining (minor to moderate discomfort, some numbness in the cooled extremity) |
| 2 | Only barely tolerated, (significant discomfort, numbness of entire cooled extremity) |
| 3 | Intolerable, (intolerable discomfort in cooled extremity, need to break off) |

**Table S4.** Shiver assessment scale for assessing tolerability of continuous-flow limb hypothermia.

| **Shiver assessment scale (SAS)** | | **Indications** |
| --- | --- | --- |
| 0 | None | No shiver |
| 1 | Mild | Fine/intermittent shivering localized to the neck and/or thorax |
| 2 | Moderate | Continuous shivering involves gross movement of the upper extremities (in addition to neck and thorax) |
| 3 | Severe | Shivering involves gross movements of the trunk and upper and lower extremities |

**Table S5.** Adverse events.

| **Adverse events** | **Number of cases** | |
| --- | --- | --- |
|  | **All grades** | **Grades 3-4** |
| Sensory neuropathy | 20 | 1 |
| Hematological |  |  |
| Neutropenia | 6 | 5 |
| Anemia | 3 | 1 |
| Renal impairment |  |  |
| Creatinine increased | 1 | 0 |
| Hypokalaemia | 1 | 1 |
| Dysuria | 1 | 0 |
| Nails |  |  |
| Nail discoloration | 3 | 0 |
| Nail infection | 2 | 0 |
| General skin toxicity  (not over region of cooling) |  |  |
| Zoster | 1 | 0 |
| Excoriations | 1 | 0 |
| Rashes | 9 | 0 |
| Erythema | 2 | 0 |
| Dermatitis | 4 | 1 |
| Skin reaction | 2 | 0 |
| Arthralgia/myalgia | 10 | 1 |
| Head and brain |  |  |
| Headache | 4 | 0 |
| Fatigue | 10 | 0 |
| Dizziness | 3 | 0 |
| Insomnia | 2 | 0 |
| Nose, mouth and throat |  |  |
| Gingivitis | 1 | 0 |
| Toothache | 1 | 1 |
| Oral mucositis | 2 | 0 |
| Dysgeusia | 1 | 0 |
| Pharyngitis | 5 | 0 |
| Respiratory |  |  |
| Upper respiratory tract infection | 8 | 0 |
| Dyspnea | 1 | 0 |
| Cough | 7 | 0 |
| Gastrointestinal |  |  |
| Diarrhea | 5 | 0 |
| Constipation | 3 | 0 |
| Vomiting | 1 | 0 |
| Dyspepsia | 1 | 0 |
| Reflux | 2 | 0 |

**Table S6.** Absolute changes (from baseline NCS) in sensory nerve conduction amplitude and velocity at one, three and six months after the start of chemotherapy in 18 breast cancer patients. Percentage changes are shown in brackets.

| Sensory amplitude | **δ (1 month)** | | | **δ (3 months)** | | | **δ (6 months)** | | |
| --- | --- | --- | --- | --- | --- | --- | --- | --- | --- |
|  | **Cooled**  **μV**  **(%)** | **Control**  **μV**  **(%)** | ***p*-value** | **Cooled**  **μV**  **(%)** | **Control**  **μV**  **(%)** | ***p*-value** | **Cooled**  **μV**  **(%)** | **Control**  **μV**  **(%)** | ***p*-value** |
| **Sural** | -1.0  (-6.4) | 0.3  (-0.6) | 0.29  (0.22) | -3.7  (-18.8) | -3.6  (-19.8) | 0.92  (0.81) | -3.3  (-19.9) | -4.7  (-25.8) | 0.18  (0.16) |
| **Superficial peroneal** | -4.7  (-12.4) | -3.1  (-10.0) | 0.24  (0.65) | -7.1  (-30.5) | -7.3  (-31.7) | 0.91  (0.78) | -8.7  (-36.2) | -7.9  (-34.4) | 0.44  (0.63) |
| **Saphenous** | -0.6  (-0.7) | -0.4  (0.01) | 0.70  (0.93) | -1.2  (-18.1) | -1.5  (-27.6) | 0.28  (0.17) | -1.5  (-24.3) | -1.6  (-25.2) | 0.68  (0.87) |
| **Medial plantar** | -2.7  (-13.7) | -3.0  (-24.0) | 0.69  (0.35) | -7.0  (-54.8) | -7.3  (-55.8) | 0.62  (0.83) | -7.8  (-59.0) | -8.0  (-59.4) | 0.77  (0.90) |
| **Lateral plantar** | -0.01  (-11.6) | -1.0  (-17.1) | 0.13  (0.29) | -2.0  (-37.8) | -2.4  (-41.9) | 0.25  (0.59) | -2.4  (-51.6) | -2.5  (-53.9) | 0.39  (0.44) |
| Sensory velocity | **Cooled**  **m/s**  **(%)** | **Control**  **m/s**  **(%)** | ***p*-value** | **Cooled**  **m/s**  **(%)** | **Control**  **m/s**  **(%)** | ***p*-value** | **Cooled**  **m/s**  **(%)** | **Control**  **m/s**  **(%)** | ***p*-value** |
| **Sural** | 2.7  (5.9) | 1.3  (4.0) | 0.68  (0.94) | 0.4  (2.2) | -0.8  (-0.7) | 0.85  (0.68) | 3.7  (9.3) | 4.4  (9.8) | 0.68  (0.82) |
| **Superficial peroneal** | 2.9  (6.7) | -1.8  (-2.6) | 0.19  (0.18) | 0.9  (2.4) | -0.9  (-1.2) | 0.38  (0.37) | 5.8  (11.3) | 5.8  (10.4) | 0.97  (0.87) |
| **Saphenous** | 0.2  (1.9) | 5.8  (14.9) | **0.03**  **(0.01)** | 0.1  (2.4) | 1.5  (6.3) | 0.59  (0.52) | 5.8  (13.6) | 8.8  (22.2) | 0.18  (0.09) |
| **Medial plantar** | 0.4  (1.0) | -2.3  (-3.9) | 0.25  (0.23) | -4.2  (-6.9) | -5.5  (-9.5) | 0.54  (0.44) | -3.4  (-5.2) | -2.7  (-4.5) | 0.83  (0.89) |
| **Lateral plantar** | 1.5  (3.0) | 1.6  (3.6) | 0.89  (0.94) | -2.7  (-4.5) | 0.1  (0.9) | 0.12  (0.11) | -5.5  (-3.2) | 0.08  (0.5) | 0.27  (0.30) |

**Table S7.** Absolute changes (from baseline NCS) in motor nerve conduction amplitude and velocity at one, three and six months after the start of chemotherapy in 18 breast cancer patients. Percentage changes are shown in brackets.

| Motor amplitude | **δ (1 month)** | | | **δ (3 months)** | | | **δ (6 months)** | | |
| --- | --- | --- | --- | --- | --- | --- | --- | --- | --- |
|  | **Cooled**  **mV**  **(%)** | **Control**  **mV**  **(%)** | ***p*-value** | **Cooled**  **mV**  **(%)** | **Control**  **mV**  **(%)** | ***p*-value** | **Cooled**  **mV**  **(%)** | **Control**  **mV**  **(%)** | ***p*-value** |
| **EDB (Ankle)** | -0.1  (5.7) | -0.1  (-0.4) | 0.87  (0.40) | -0.9  (-15.2) | -0.7  (-11.6) | 0.67  (0.72) | -0.4  (-2.7) | -0.7  (-16.2) | 0.17  (0.09) |
| **EDB (Below fib head)** | -0.1  (4.7) | -0.1  (1.1) | 0.87  (0.63) | -0.8  (-15.3) | -0.6  (-13.1) | 0.62  (0.78) | -0.4  (-2.1) | -0.7  (-18.3) | 0.23  (0.07) |
| **EDB (Above fib head)** | -0.2  (3.0) | -0.03  (1.9) | 0.59  (0.88) | -0.8  (-16.7) | -0.6  (-12.9) | 0.60  (0.66) | -0.4  (-4.3) | -0.7  (-18.7) | 0.26  (0.10) |
| **AH (Ankle)** | -0.6  (-4.3) | -0.7  (-3.0) | 0.89  (0.80) | -0.9  (-6.9) | -1.6  (-10.6) | 0.35  (0.41) | -0.8  (-7.2) | -1.7  (-10.7) | 0.17  (0.41) |
| **AH (Knee)** | 0.3  (3.6) | 0.2  (4.7) | 0.85  (0.85) | -0.1  (-0.2) | -0.8  (-5.5) | 0.08  (0.20) | -0.2  (-2.3) | -1.6  (-12.7) | **0.04**  (0.07) |
| Motor velocity | **Cooled**  **m/s**  **(%)** | **Control**  **m/s**  **(%)** | ***p*-value** | **Cooled**  **m/s**  **(%)** | **Control**  **m/s**  **(%)** | ***p*-value** | **Cooled**  **m/s**  **(%)** | **Control**  **m/s**  **(%)** | ***p*-value** |
| **EDB (Below fib head)** | 1.3  (2.5) | 0.92  (1.7) | 0.62  (0.59) | -1.2  (-2.5) | -0.9  (-1.7) | 0.49  (0.52) | 1.3  (3.0) | 1.1  (2.4) | 0.85  (0.82) |
| **EDB (Above fib head)** | 1.0  (2.1) | 2.3  (6.2) | 0.58  (0.38) | -2.8  (-5.0) | 0.14  (1.11) | 0.2  (0.2) | -0.7  (-0.2) | 3.7  (8.7) | 0.08  (0.07) |
| **AH (Knee)** | 1.2  (2.3) | 0.7  (1.8) | 0.73  (0.86) | -2.9  (-6.0) | -1.9  (-3.8) | 0.44  (0.42) | -0.4  (-0.6) | -0.2  (-0.2) | 0.86  (0.87) |

# Supplementary Figures

**
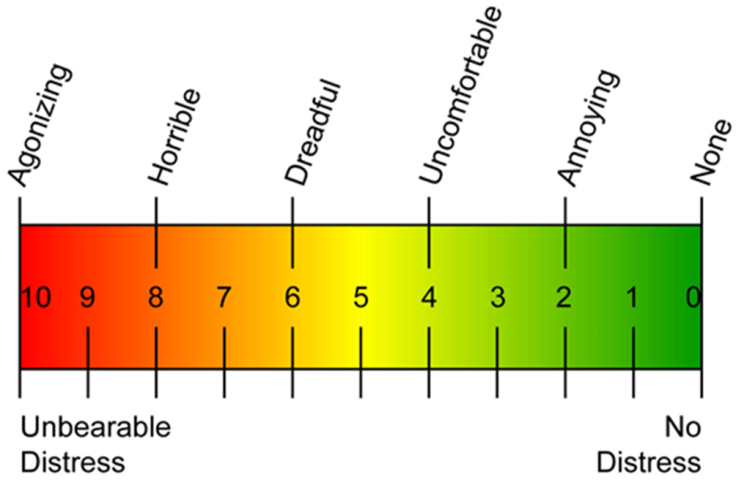
**

**Figure S1.** Various measures/scales for the assessment of limb hypothermia tolerability in breast cancer patients including the visual analogue pain scale, shown here, subjective tolerance scale (Table S3) and shivering assessment scale (Table S4). Composite tolerability scale is the combined measure of the previous three scales and is used to determine dose limiting tolerability.


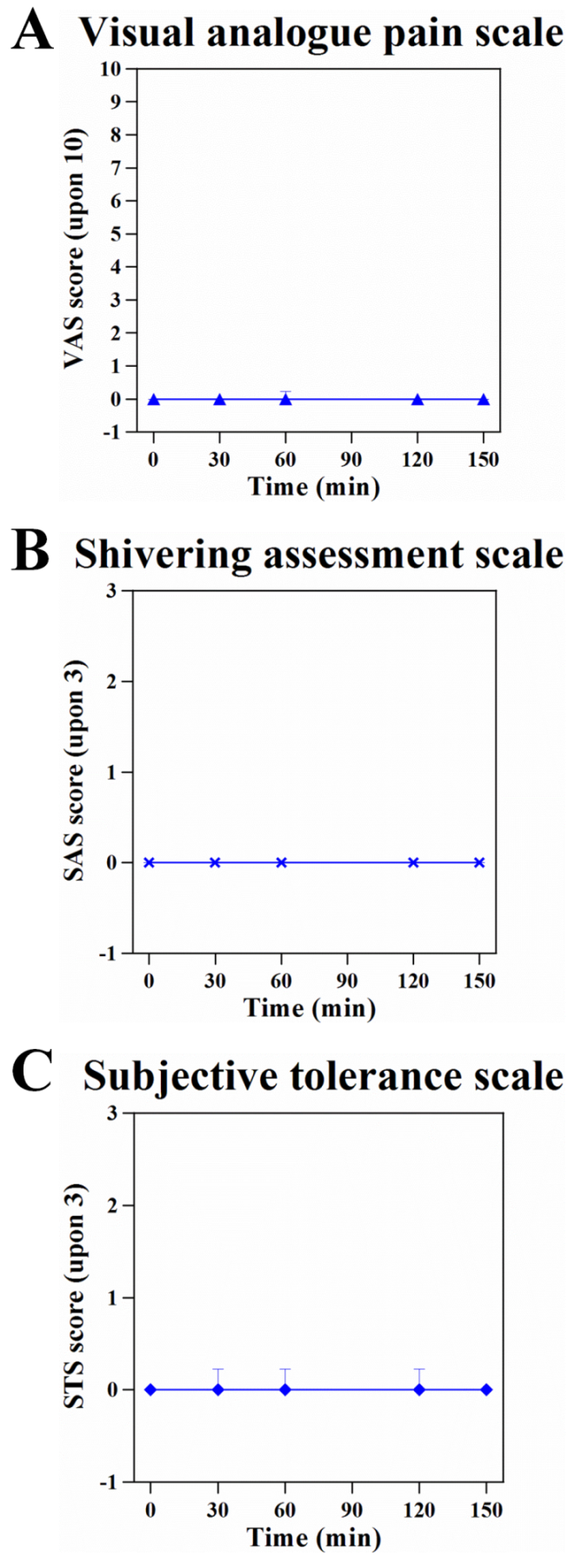


**Figure S2.** Trend of tolerability scores reported throughout the duration of limb hypothermia over 218 cycles. Visual analogue scale (A), subjective tolerance scale (B) and shivering assessment scale (C) are depicted.


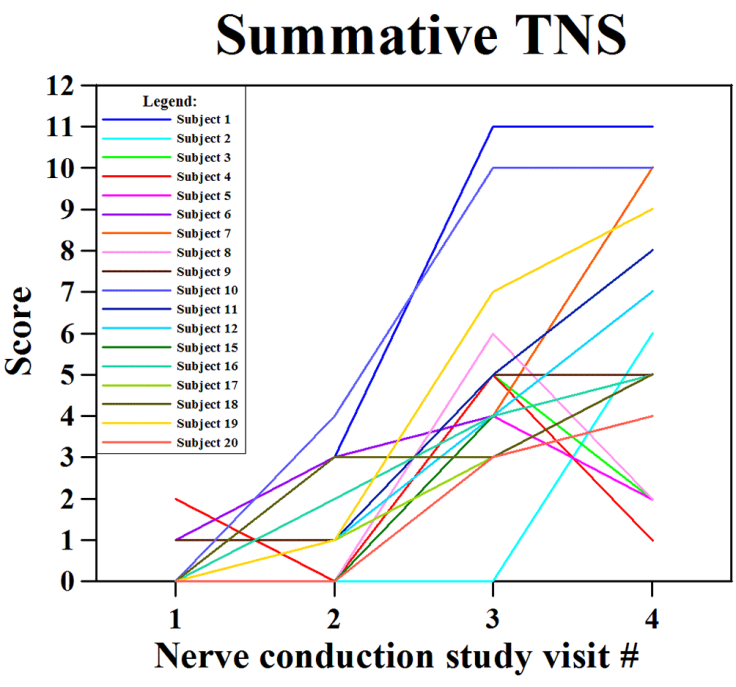


**Figure S3.** Summative TNS score trends of each patient over the four NCS visits. 10 out of 20 patients (50%) had a TNS that increased between the last two visits, indicating worsened neuropathic symptoms and nerve function. 4 out of 20 patients (20%) showed a TNS that decreased between the last two visits, indicating lessened neuropathic symptoms and improved nerve function. 3 out of 20 patients (15%) had no change in TNS between the last two visits. One patient could not complete the TNS on the last visit due to technical difficulties.

**References**

Karandreas, N., Papatheodorou a Fau - Triantaphilos, I., Triantaphilos I Fau - Mavridis, M., Mavridis M Fau - Lygidakis, C., and Lygidakis, C. (1995). Sensory nerve conduction studies of the less frequently examined nerves. *Electromyogr Clin Neurophysiol* 35**,** 169-173.

Ping Ng, K.W., Ong, J.J.Y., Nyein Nyein, T.D., Liang, S., Chan, Y.C., Lee, K.O., and Wilder-Smith, E.P. (2013). EMLA-Induced Skin Wrinkling for the Detection of Diabetic Neuropathy. *Frontiers in Neurology* 4**,** 126.
